# Lineage Dynamics and Risk Factors Underlying Serotype 4 Invasive Pneumococcal Disease in Spain

**DOI:** 10.1093/infdis/jiag326

**Published:** 2026-06-30

**Authors:** Covadonga Pérez-García, Joaquin Llorente, Maria Elena Aguirre Alastuey, Mirella Llamosí, Ruth Gil-Prieto, Gabriel Laghlali, Farah El-Ayache, Vivian Yan, Michael Schotsaert, Jorge del Diego-Salas, Jose Miguel Cisneros, Adolfo García-Sastre, Mirian Domenech, Julio Sempere, Jose Yuste

**Affiliations:** Spanish Pneumococcal Reference Laboratory, National Center for Microbiology, Instituto de Salud Carlos III, Madrid, Spain; CIBER de Enfermedades Respiratorias (CIBERES), Instituto de Salud Carlos III, Madrid, Spain; Clinical Unit of Infectious Diseases, Microbiology and Parasitology, Hospital Universitario Virgen del Rocío, Seville, Spain; Doctoral Program in Biomedical Sciences and Public Health, Spanish University of Distance Education, Madrid, Spain; Internal Medicine Service, Hospital Universitario Virgen de Valme, Seville, Spain; Spanish Pneumococcal Reference Laboratory, National Center for Microbiology, Instituto de Salud Carlos III, Madrid, Spain; CIBER de Enfermedades Respiratorias (CIBERES), Instituto de Salud Carlos III, Madrid, Spain; Department of Medical Specialties and Public Health, University Rey Juan Carlos, Alcorcón, Madrid, Spain; Department of Microbiology, Icahn School of Medicine at Mount Sinai, New York, New York, USA; Global Health and Emerging Pathogens Institute, Icahn School of Medicine at Mount Sinai, New York, New York, USA; Department of Microbiology, Icahn School of Medicine at Mount Sinai, New York, New York, USA; Global Health and Emerging Pathogens Institute, Icahn School of Medicine at Mount Sinai, New York, New York, USA; Department of Microbiology, Icahn School of Medicine at Mount Sinai, New York, New York, USA; Global Health and Emerging Pathogens Institute, Icahn School of Medicine at Mount Sinai, New York, New York, USA; Graduate School of Biomedical Sciences, Icahn School of Medicine at Mount Sinai, New York, New York, USA; Department of Microbiology, Icahn School of Medicine at Mount Sinai, New York, New York, USA; Global Health and Emerging Pathogens Institute, Icahn School of Medicine at Mount Sinai, New York, New York, USA; The Icahn Genomics Institute, Icahn School of Medicine at Mount Sinai, New York, New York, USA; Marc and Jennifer Lipschultz Institute for Precision Immunology, Icahn School of Medicine at Mount Sinai, New York, New York, USA; Department of Immunology and Immunotherapy, Icahn School of Medicine at Mount Sinai, New York, New York, USA; Health and Consumption Department, General Directorate of Public Health and Pharmaceutical Management of Andalusia, Seville, Spain; Health promotion and prevention, Spanish Ministry of Health, Madrid, Spain; Clinical Unit of Infectious Diseases, Microbiology and Parasitology, Hospital Universitario Virgen del Rocío, Seville, Spain; CIBER de Enfermedades Infecciosas (CIBERINFEC), Instituto de Salud Carlos III, Madrid, Spain; Department of Microbiology, Icahn School of Medicine at Mount Sinai, New York, New York, USA; Global Health and Emerging Pathogens Institute, Icahn School of Medicine at Mount Sinai, New York, New York, USA; The Icahn Genomics Institute, Icahn School of Medicine at Mount Sinai, New York, New York, USA; Division of Infectious Diseases, Department of Medicine, Icahn School of Medicine at Mount Sinai, New York, New York, USA; The Tisch Cancer Institute, Icahn School of Medicine at Mount Sinai, New York, New York, USA; Department of Pathology, Molecular and Cell-Based Medicine, Icahn School of Medicine at Mount Sinai, New York, New York, USA; Spanish Pneumococcal Reference Laboratory, National Center for Microbiology, Instituto de Salud Carlos III, Madrid, Spain; CIBER de Enfermedades Respiratorias (CIBERES), Instituto de Salud Carlos III, Madrid, Spain; Spanish Pneumococcal Reference Laboratory, National Center for Microbiology, Instituto de Salud Carlos III, Madrid, Spain; CIBER de Enfermedades Respiratorias (CIBERES), Instituto de Salud Carlos III, Madrid, Spain; Spanish Pneumococcal Reference Laboratory, National Center for Microbiology, Instituto de Salud Carlos III, Madrid, Spain; CIBER de Enfermedades Respiratorias (CIBERES), Instituto de Salud Carlos III, Madrid, Spain

**Keywords:** serotype 4, ST15063, GPSC162, tobacco, H3N2

## Abstract

**Background:**

The emergence of vaccine-covered serotypes causing invasive pneumococcal disease (IPD) is a serious concern worldwide.

**Methods:**

We characterized all national serotype 4 IPD isolates from children and adults received at the Spanish Pneumococcal Reference Laboratory (2009–2024).

**Results:**

We investigated the unexpected rise of serotype 4 causing IPD in young adults after the COVID-19 pandemic, affecting the elderly population in recent years. Epidemiological and genomic analysis confirmed that the rise started as an abrupt cluster of cases in Seville (Andalusia) in the year 2022 due to the ST15063 within the GPSC12 lineage. This outbreak initially affected nonvaccinated young individuals associated with high rates of tobacco smoking, alcohol, and inhaled drugs followed by a general distribution through the country in the following years that affected adults ≥65 years old. Moreover, ST15063 serotype 4 strains displayed enhanced infection rates of human lung cells that significantly increased in the presence of cigarette smoke exposure and by influenza A(H3N2) coinfection.

**Conclusions:**

These findings highlight the need for targeted vaccination strategies, molecular surveillance, and focused interventions in high-risk populations.

## Teaser

The increase of pneumococcal serotype 4 in adults is important for public health, as it is covered by PCV20 but absent in PCV21. Identifying target populations and risk factors is essential to design effective preventive strategies against pneumococcal disease overall.

Invasive pneumococcal disease (IPD) remains a significant public health concern worldwide. In Spain, the use of pneumococcal conjugate vaccines (PCVs) in children started in 2001 with 7-valent PCV (PCV7), which was replaced in 2010 with 13-valent PCV (PCV13) in the private market and finally introduced in the national pediatric calendar in 2016 with high coverage rates >95%. The use of these vaccines clearly reduced the burden of disease including antibiotic resistance with a clear impact in adult IPD due to their herd-immunity effects [1–4]. However, serotype replacement by non-PCV13 serotypes has been increasingly reported in recent years, especially serotype 8 [2, 5, 6], and since the coronavirus disease 2019 (COVID-19) pandemic, cases by PCV13 serotypes such as serotype 3 have also spiked in both adult and pediatric populations [5, 7].

While serotype 4 has historically been uncommon in adult IPD, recent surveillance data indicate a notable rise in IPD cases caused by this vaccine serotype, especially among Spanish adults aged 18–64 years, ranking from ninth in 2019 to the third cause of IPD by 2022–2023 [5]. This pattern has also been observed in other countries; for example, in the United Kingdom (UK), IPD by serotype 4 was scarce in previous seasons, but cases surged during 2022–2023 in young adults [8]. In Canada, serotype 4 was also one of the main causes of the resurgence of IPD cases in adults after the COVID-19 pandemic [9, 10]. However, other countries only have linked serotype 4 cases to specific high-risk groups, such as individuals experiencing homelessness or opioid use [11–13], or specific outbreaks in workers such as in shipyards in Northern Europe [14–17].

Given the changing epidemiology of serotype 4 and its absence in certain adult vaccines, such as 21-valent PCV (PCV21), understanding its recent emergence is essential to update vaccine policy and IPD prevention strategies. This is a national epidemiological surveillance study of serotype 4 from 2004 through 2024 including molecular characterization, clinical analysis of hospitalized patients, and host–pathogen interaction with human lung cells evaluating the impact of cigarette smoke exposure and influenza virus coinfections.

## MATERIALS AND METHODS

### Study Design

For this study, we considered all serotype 4 isolates received at the laboratory covering the period 2009–2024 (N = 827). IPD trends were analyzed for different age groups covering the pediatric population (<2 years, 2–4 years, 5–17 years) and adult population (18–64 years, ≥65 years). Isolates from 2009–2024 were divided in different vaccine periods (pre-PCV13, 2009; early PCV13, 2010–2012; middle PCV13, 2013–2016; late PCV13, 2017–2019; COVID-19, 2020–2021; reopening, 2022–2023; new PCVs, 2024). Serotyping was performed using Quellung reaction, dot blot assay using specific antisera, and/or polymerase chain reaction–capsular sequence typing [2, 5]. Additional information is indicated in the Supplementary Methods.

### Whole Genome Sequencing and Bioinformatic Analysis

For the whole genome sequencing (WGS) study, we sequenced 60%–96% of isolates from years 2009 (pre-PCV13), 2015 (middle PCV13), 2018–2019 (late PCV13), 2021 (COVID-19), 2022–2023 (reopening), and 2024 (new PCVs). WGS analysis was conducted using the Bactopia Nextflow pipeline [18]. Protocols are detailed in the Supplementary Methods.

### Clinical Data From Patients Hospitalized With IPD

All clinical data were anonymized, and the study was approved by the Ethics Committee of the Hospital Universitario Virgen del Rocío (ENISE4, SICEIA-2025-002346). Demographic variables, comorbidities, risk factors, vaccination status, and mortality were recorded. Full description of data processing is included in the Supplementary Methods.

### Infection of Human Nasopharyngeal and Lung Epithelial Cells

Infection assays of Detroit 562 and A549 cells were performed using different genotypes of pneumococcal isolates of serotype 4. Lung cells were infected in the presence or absence of cigarette smoke extract. For coinfection experiments between serotype 4 (sequence type [ST] 15063 and ST205) and influenza virus, we used influenza A/New Caledonia/20/1999 (H1N1 subtype) or influenza A/Darwin/9/2021 (H3N2 subtype). All experimental conditions and protocols are described in the Supplementary Methods.

### Statistical Analysis

Statistical analysis is thoroughly described in the Supplementary Methods. For incidence rate ratios (IRRs), we used Poisson regression models. Stata version 14 and R 4.5.1 were used for incidence analysis and correlation between comorbidities and genotypes. For experimental procedures, we used GraphPad InStat software version 8.0.

## RESULTS

### Expansion of Serotype 4 Cases in Young Adults Since the COVID-19 Pandemic

Serotype 4 cases in children were scarce even before PCV13 introduction (8 cases in 2009) (Figure 1*A* and 1*B*). IPD cases diminished after vaccine introduction, showing only 2 IPD cases by serotype 4 in the late PCV13 period (2017–2019), considering all pediatric age groups. Lack of serotype 4 cases in children was reported during COVID-19, although a moderate upsurge was found during 2022–2024 (Figure 1*A* and 1*B*).

**Figure 1.**
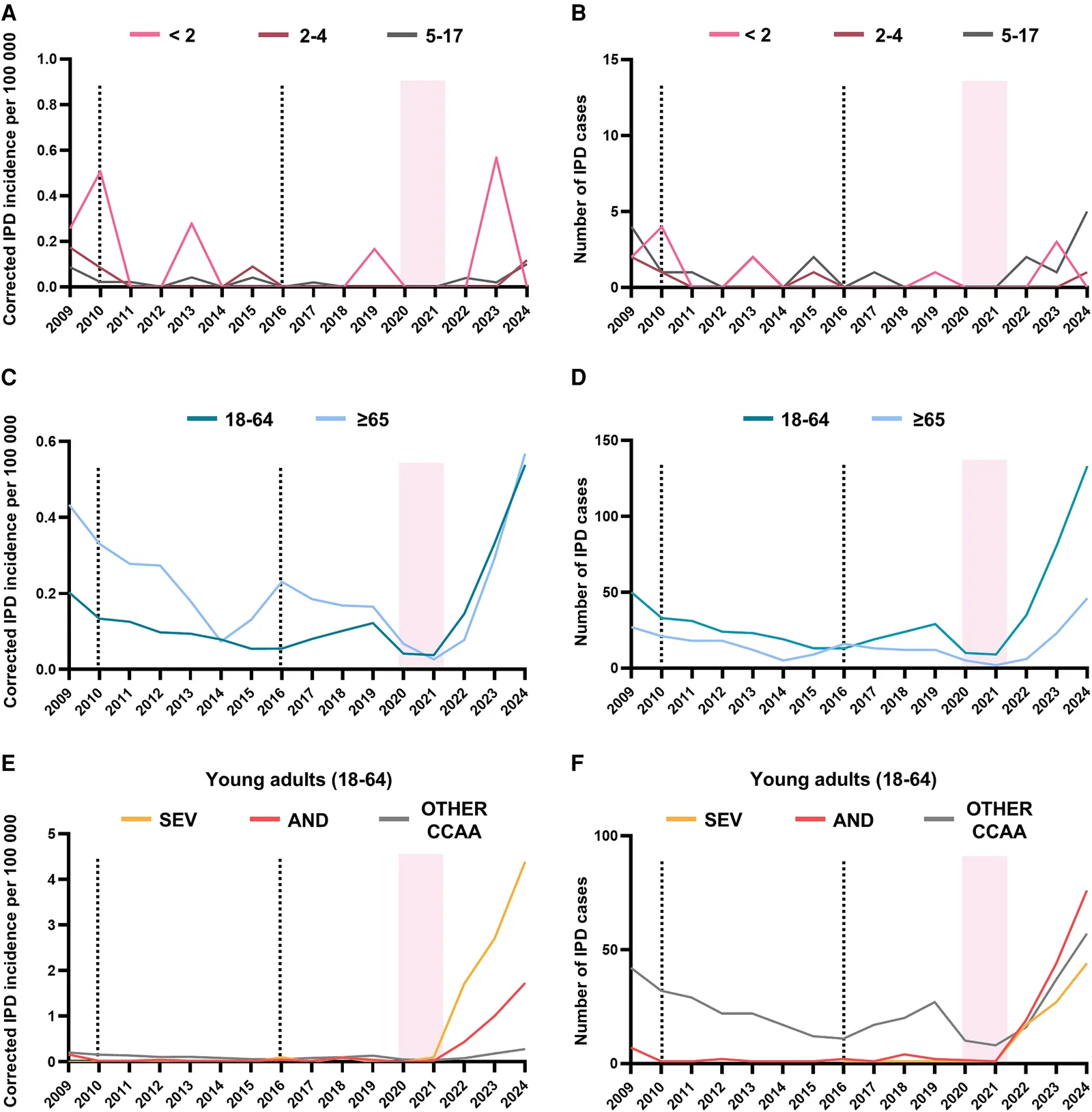
Corrected annual incidence and number of invasive pneumococcal disease (IPD) cases of serotype 4 in Spain in pediatric and adult populations during 2009–2024. IPD incidence rates (*A*) and cases (*B*) for the pediatric populations <2 years, 2–4 years, and 5–17 years. IPD incidence rates (*C*) and cases (*D*) for adult populations 18–64 years old and ≥65 years old. IPD incidence rates (*E*) and cases (*F*) for young adults (18–64 years) living in the subregion-province of Seville (SEV), the region-autonomous community (CCAA) of Andalusia (AND), or other Spanish regions (OTHER CCAA). Vertical lines represent the years when 13-valent pneumococcal conjugate vaccine was available in the private market (2010) and was added to the national childhood immunization calendar (2016). Shading represents the COVID-19 years.

In adults, serotype 4 IPD declined with the introduction of PCV13 in children (Figure 1*C* and 1*D*), confirming herd-protection immunity against this serotype. During the middle vaccine period (2013 vs 2009), incidence rates were significantly lower for serotype 4 (IRR, 0.25 [95% confidence interval {CI], .17–.37] for the age group 18–64 years and IRR, 0.36 [95% CI, .22–.58] for ≥65 years). These results were also confirmed when assessing the late PCV13 period (2017–2019) versus 2009 (Figure 1*C* and 1*D*, Table 1). During COVID-19 (2020–2021), we observed a reduction of cases due to the implementation of nonpharmacological measures (Figure 1*C* and 1*D*, Table 1). Remarkably, after the end of containment measures, serotype 4 IPD cases spiked in all adult populations, but specifically in young adults (Figure 1*C* and 1*D*). The upward trend began in 2022, with reported cases increasing from 9 in 2021 to 35 in 2022, 81 in 2023, and 133 in 2024 (Figure 1*D*). This expansion of serotype 4 cases in young adults was significant when comparing the last 3 full epidemiological years (2022–2024) versus all the other vaccine periods, even before the introduction of PCV13 (2022–2024 vs 2009: IRR, 1.63 [95% CI, 1.24–2.28]; 2022–2024 vs 2017–2019: IRR, 3.36 [95% CI, 2.59–4.37]; 2022–2024 vs 2020–2021: IRR, 8.57 [95% CI, 5.38–13.67]) (Figure 1*C* and 1*D*, Table 1). In adults ≥65 years old, this increase was also observed, being especially relevant in the last 2 full epidemiological years (2022–2024 vs 2020–2021: IRR, 6.77 [95% CI, 3.12–14.69]; 2022–2024 vs 2017–2019: IRR, 1.83 [95% CI, 1.24–2.27]) (Figure 1*C* and 1*D*, Table 1).

**Table 1.** Number of Cases, Corrected Incidence (per 100 000), and Incidence Rate Ratios of Serotype 4 Invasive Pneumococcal Disease in the Different Vaccine Periods

Age Group	2009		2010–2012		2013–2016		IRR 2013–2016 vs 2009	95% CI	IRR 2017–2019 vs 2009	95% CI	IRR 2020–2021 vs 2017–19	95% CI
	Cases	Corrected Incidence	Cases	Corrected Incidence	Cases	Corrected Incidence						
18–64 y	50	0.20	88	0.12	49	0.05	**0**.**25**	**.17–.37**	**0**.**50**	**.35–.72**	**0**.**39**	**.24–.64**
≥65 y	27	0.43	57	0.29	42	0.15	**0**.**36**	**.22–.58**	**0**.**40**	**.24–.66**	**0**.**27**	**.12–.40**
All adults	77	0.25	415	0.16	91	0.07	**0**.**30**	**.22–.40**	**0**.**47**	**.35–.63**	**0**.**35**	**.23–.53**

	2017–2019		2020–2021		2022–2024		IRR 2022–2024 vs 2009	95% CI	IRR 2022–2024 vs 2020–2021	95% CI	IRR 2022–2024 vs 2017–2019	95% CI
	Cases	Corrected incidence	Cases	Corrected incidence	Cases	Corrected incidence						
18–64 y	72	0.10	19	0.04	249	0.34	**1**.**63**	**1.24–2.28**	**8**.**57**	**5.38–13.67**	**3**.**36**	**2.59–4.37**
≥65 y	37	0.17	7	0.05	75	0.32	0.73	.50–1.07	**6**.**77**	**3.12–14.69**	**1**.**83**	**1.24–2.27**
All adults	109	0.12	26	0.04	312	0.32	**1**.**29**	**1.02–1.63**	**7**.**78**	**5.22–11.61**	**2**.**73**	**2.20–3.40**

Corrected incidence is per 100 000. Statistically significant IRRs are highlighted in bold. Abbreviations: CI, confidence interval; IRR, incidence rate ratio.

We observed that most of these cases in young adults belonged to the same Spanish geographical autonomous community (CCAA)/region (Andalusia) and specifically, the province/subregion of Seville (Figure 2, Supplementary Figure 1). In the last 3 years, 19 of 35 cases in 2022, 44 of 81 cases in 2023, and 76 of 133 cases in 2024 appeared in Andalusia (and 57%–89% of them in Seville) (Figure 1*E* and 1*F*, Figure 2). By 2024, serotype 4 IPD cases had spread across all Spanish regions, although Andalusia/Seville remained the main hotspot. Cases were also increasingly observed among adults aged ≥65 years, indicating dissemination into the older population (Figure 1*C*–*F*, Figure 2).

**Figure 2.**
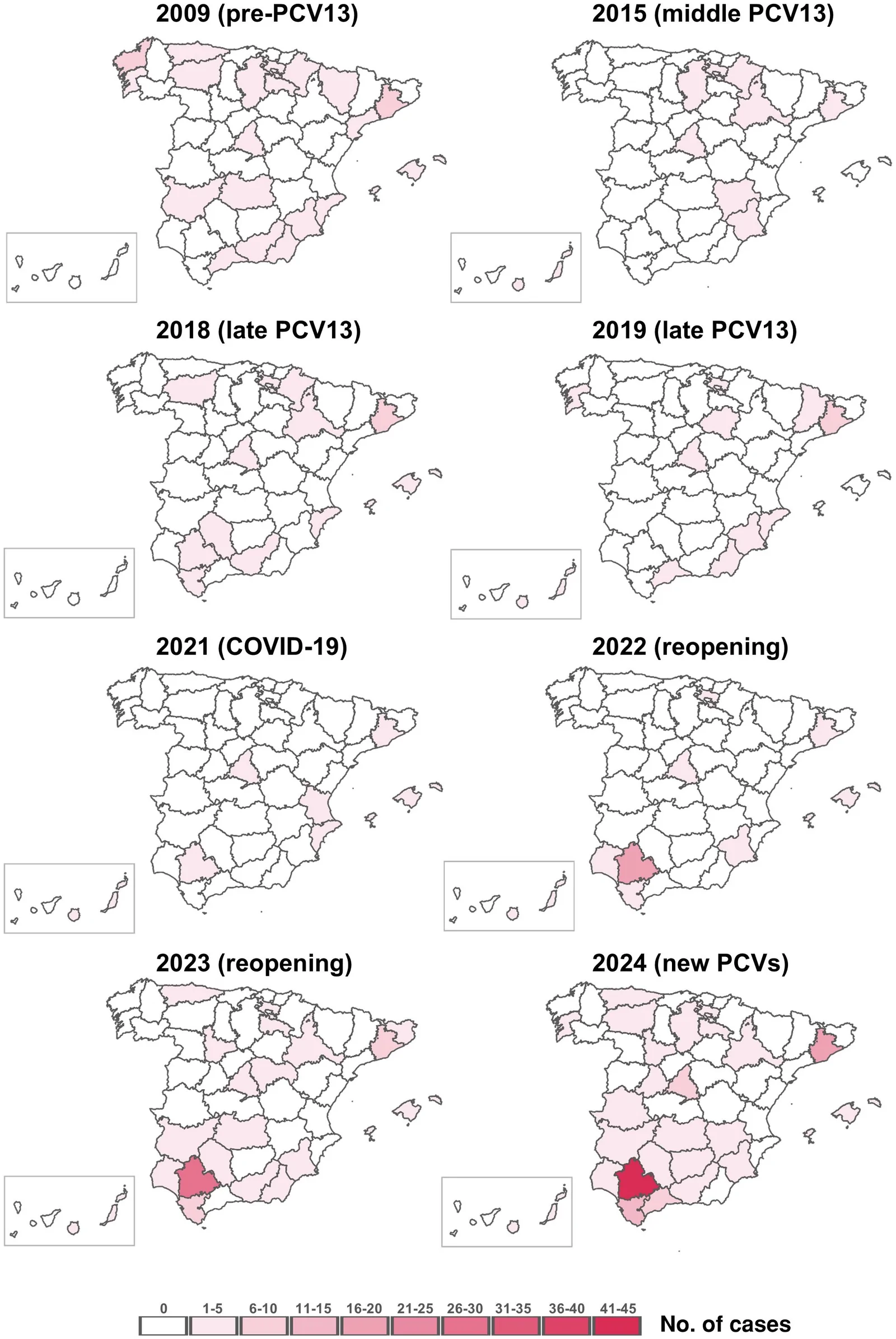
Geographic expansion of serotype 4 in young adults (18–64 years old) in Spain. The map shows the number of invasive pneumococcal disease cases notified to the Spanish Pneumococcal Reference Laboratory by subregion and by epidemiological year (from January to December). Seville is located in Andalusia, which is in the south of Spain, and the location is indicated in Supplementary Figure 1. Abbreviations: COVID-19, coronavirus disease 2019; PCVs, pneumococcal conjugate vaccines; PCV13, 13-valent pneumococcal conjugate vaccine.

### ST15063/CC801/GPSC162 Dominates Serotype 4 in Recent Spread

WGS was performed in more than 60% of all serotype 4 isolates from the period 2009–2024 (Supplementary Table 1). Evaluating all periods, we observed up to 11 different lineages, although 4 lineages encompassed most cases being GPSC162 (n = 271), GPSC27 (n = 66), CC1866 (with not assigned Global Pneumococcal Sequence Cluster [GPSC]) (n = 17), and GPSC70 (n = 14) (Figure 3*A*). In 2009, most isolates belonged to GPSC27 (56.9%) containing ST205, ST247, and ST246 (Figure 3), but since PCV13 introduction, IPD cases by this lineage diminished. In contrast, we observed a remarkable evolution of GPSC162 associated with the rise of serotype 4, with very few cases in the pre-PCV13 and middle vaccine periods. However, during the late PCV13 period, GPSC162 became the most frequent cause of IPD among serotype 4, although with a low burden of disease (Figure 3*A*). Nevertheless, after the uplift of COVID-19 containment measures, there was an outburst of serotype 4 cases in Spain, mainly driven by ST15063 within GPSC162 (63% of sequenced isolates during 2022–2024) (Figure 3). Moreover, most Andalusia isolates of serotype 4 from 2022–2024 belonged to ST15063 (94.8%), and the same distribution was found for most Seville isolates (97.1%) (Supplementary Table 1). More than 97% of Andalusia isolates and all Seville isolates were closely related, being grouped within the ST15063 clade or a very closely related clade of the single-locus variant ST801 (Figure 4). ST15063 and ST801 were more distantly related to other STs within GPSC162 such as ST1222 and ST13022, which originated from other Spanish regions (Figure 4, Supplementary Figure 2). Serotype 4 isolates from other Spanish regions were spread along the phylogenetic trees, but we observed a highly related cluster from Andalucia and specifically from Seville (3–15 nucleotide differences between Seville ST15063 isolates), suggesting clonal differences between Andalusia and other regions (Figure 4) and pneumococcal outbreak dynamics. Since the expansion of serotype 4 in 2022–2024, IPD cases by serotype 4 in other regions presented more lineage diversity (Figure 4, Supplementary Figure 2). The lineages circulating in older adults between 2022 and 2024 are similar to those found in young adults. Of 58 isolates belonging to adults ≥65 years old, 78% (45/58) belonged to GPSC162 and 53% (31/58) were associated with ST15063/CC801/GPSC162 (Supplementary Table 1).

**Figure 3.**
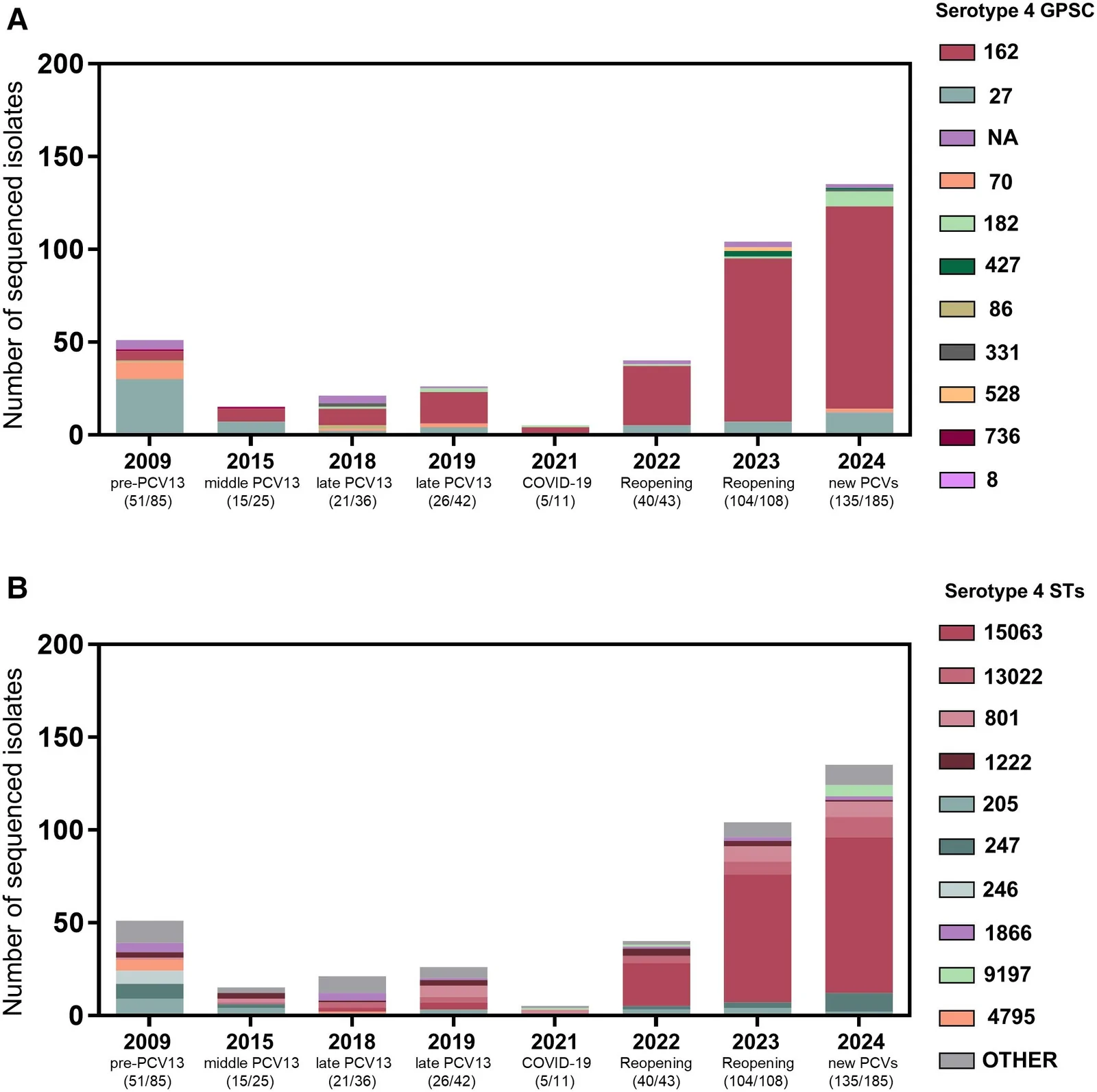
Trends in the distribution of lineages among serotype 4 isolates across different vaccine periods covering 2009–2024. *A*, Serotype 4 GPSC distribution by year. *B*, Serotype 4 sequence type distribution by year. Abbreviations: COVID-19, coronavirus disease 2019; GPSC, Global Pneumococcal Sequence Cluster; PCVs, pneumococcal conjugate vaccines; PCV13, 13-valent pneumococcal conjugate vaccine; ST, sequence type.

**Figure 4.**
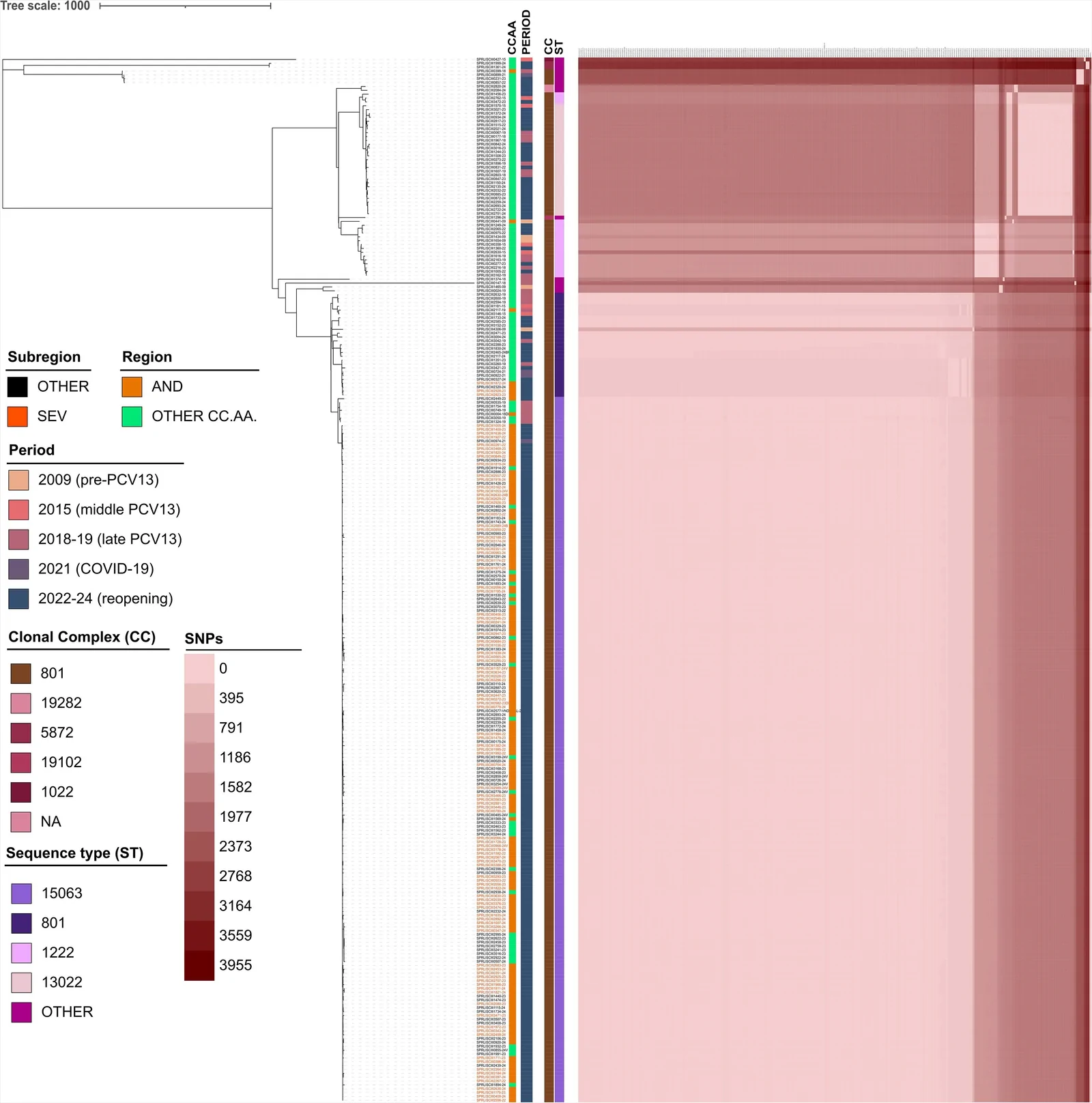
Phylogenetic tree of GPSC162 in Spain. The phylogenetic tree was obtained using the snippy, snippy-core, and Gubbins steps described in the Materials and Methods, and groups genomes into different clades based on the SNPs against the reference (GCA_09128732). Heatmap displaying differences in the SNPs, using GCA_09128732 as a reference. The geographic region was depicted (Andalusia vs other regions), and Seville isolates are highlighted in orange. Annotated data are described in the legend. Abbreviations: AND, Andalusia; CCAA, autonomous community; COVID-19, coronavirus disease 2019; PCV13, 13-valent pneumococcal conjugate vaccine; SEV, Seville; SNP, single-nucleotide polymorphism; ST, sequence type.

In general, the presence of mobile genetic elements (MGEs) was scarce in serotype 4 (Supplementary Table 1). Only 27 isolates showed the presence of MGEs related to resistance determinants leading to macrolide resistance [*erm*(B), *mef*(A), and *msr*(D)], chloramphenicol resistance (*cat*), and tetracycline resistance [*tet*(M)] (Supplementary Table 1). Most cases with MGEs were related to the presence of Tn*5252* family transposon. The presence of prophages was also rare (n = 13), with the majority belonging to the PPH010 family of pneumococcal prophagues (PPHs) (Supplementary Table 1) [19]. In addition, we found differences between lineages in the presence of pilus type I, as none of the GPSC162 or CC1866 (GPSC not assigned) isolates contained the pilus islet 1 (PI-1) encoding pilus type 1. On the contrary, all GPSC27 and GPSC70 isolates, and most GPSC182 isolates, contained the PI-1 (Supplementary Table 1). Notably, all GPSC162 isolates but 2 (269/271 [99.3%]) presented a LytA mutation in the enzymatic active domain (166His→Tyr) due to a 496C→T substitution in the *lytA* gene (Supplementary Figure 3). None of the other lineages presented this mutation, except ST247/CC899/GPSC27, which had 2 LytA mutations (166His→Tyr and 171Gly→Asp), one in the enzimatic active domain and other in the linker domain (Supplementary Figure 3, Supplementary Table 1) [20].

Pangenome analysis revealed that the overall serotype 4 pangenome was very open, with nearly 40% of accessory genome (shell and cloud genes) (Supplementary Figure 4*A*). In contrast, ST15063 presented a smaller total gene count (1999 genes) and a very closed pangenome with only 5.5% of accessory genome, whereas other GPSCs and STs presented a higher total gene count and a more open pangenome (Supplementary Figure 4*A* and 4*B*).

### Serotype 4 IPD Patients in Seville Are Associated With Substance Use as a Risk Factor

We evaluated the clinical and demographic characteristics of hospitalized patients with serotype 4 IPD compared with those infected by other serotypes in Seville, Andalusia. The mean age was identical between groups, while the proportion of male patients was slightly higher in the serotype 4 group (*P* = .061). The increase of serotype 4 cases was not associated with different comorbidities such as chronic obstructive pulmonary disease, asthma, other respiratory diseases, arterial hypertension, diabetes mellitus, and dyslipidemia (Table 2).

**Table 2.** Clinical and Demographic Characteristics of Young Adult Patients From Seville, Spain, With Invasive Pneumococcal Disease (IPD) by Serotype 4 or IPD by Other Serotypes

Characteristic	Serotype 4	Other Serotype	*P* Value^a^
Available cases	85	131	
Clinical characteristics			
Age, y, mean (SD)	48 (11.4)	48 (11.5)	
Male sex	69 (81.2)	90 (68.7)	.061
Demographic characteristics			
Homelessness or penitentiary status	8 (9.4)	2 (1.5)	.**017**^b^
Substance use			
Smoking	77 (90.6)	90 (68.7)	**<**.**001**^c^
Alcohol consumption	37 (43.5)	47 (35.8)	.**036**^c^
Parenteral drug use	15 (17.6)	9 (6.9)	.**021**^c^
Inhaled drug use	32 (37.6)	21 (16.0)	**<**.**001**^c^
Polydrug use	29 (34.1)	13 (9.9)	**<**.**001**^c^
Comorbidities			
COPD	13 (15.3)	15 (11.5)	.539^c^
Asthma	6 (7.1)	7 (5.3)	.822^c^
Severe COVID-19	0 (0.0)	10 (7.6)	.**007**^b^
Other respiratory disease	5 (5.9)	11 (8.4)	.679^c^
HIV infection	6 (7.1)	9 (6.9)	1.0^c^
Immunosuppression (any cause)	5 (5.9)	35 (26.7)	**<**.**001**^c^
Corticosteroid use	7 (8.2)	34 (25.9)	.**002**^c^
Arterial hypertension	16 (18.8)	23 (17.6)	.956^c^
Diabetes mellitus	12 (14.1)	17 (13.0)	.971^c^
Dyslipidemia	10 (11.8)	19 (14.5)	.808^c^
Vaccine status			
COVID-19 vaccination	70 (82.3)	116 (88.5)	.369^b^
PCV13 or PCV13 + PPSV23	7 (8.2)	33 (25.2)	.**008**^b^
Outcome			
Exitus	5 (5.9)	9 (6.9)	.996^c^

Data are presented as No. (%) unless otherwise indicated. Values in bold indicate statistically significant results.

Abbreviations: COPD, chronic obstructive pulmonary disease; COVID-19, coronavirus disease 2019; HIV, human immunodeficiency virus; PCV13, 13-valent pneumococcal conjugate vaccine; PPSV23, 23-valent pneumococcal polysaccharide vaccine; SD, standard deviation.

^a^Primary comparison was between patients infected by serotype 4 versus patients infected by all other serotypes.

^b^Fisher exact test.

^c^Pearson χ^2^ test.

However, we observed significant differences when comparing living conditions and risk factors. Hospitalized patients by serotype 4 were more frequently homeless or imprisoned (9.4% vs 1.5%, *P* = .017). In addition, we found significant associations of serotype 4 in patients with risk factors for IPD including smoking (90.6% vs 68.7%, *P* < .001), alcohol consumption (43.5% vs 35.8%, *P* = .036), parenteral drug use (17.6% vs 6.9%, *P* = .021), inhaled drugs such as cocaine and cannabis (37.6% vs 16.0%, *P* < .001), and polydrug use (34.1% vs 9.9%, *P* < .001) (Table 2).

Conversely, hospitalized patients by other serotypes were more frequently affected by comorbidities typically associated with increased risk for IPD, such as immunosuppression of any cause (26.7% vs 5.9%, *P* < .001) and chronic corticosteroid use (25.9% vs 8.2%, *P* = .002). Patients hospitalized by other serotypes also showed a higher proportion of severe COVID-19 cases (7.6% vs 0%, *P* = .007). Finally, vaccination status differed between groups: pneumococcal vaccination (PCV13 or PCV13 + 23-valent pneumococcal polysaccharide vaccine [PPSV23]) was more common in patients with IPD by other serotypes (25.2% vs 8.2%, *P* = .008) (Table 2).

### Cigarette Smoke Exposure and H3N2 Influenza Virus Infection Increase Serotype 4 Infection in Lung Cells

We first evaluated the infectivity of different serotype 4 strains, including clinical isolates from the Andalusia (Seville) outbreak (ST15063/GPSC162) or non-outbreak-related (ST205/GPSC27). Our results confirmed that serotype 4 strains of ST15063 had an increased ability to infect lung cells than strains of ST205 and this effect was even higher in the presence of cigarette smoke extract (Figure 5*A* and 5*B*). Moreover, ST15063 had increased adherence to the upper and lower respiratory tract than other STs within GPSC162, such as ST13022 (Supplementary Figure 5). Coinfection experiments with influenza viruses confirmed that serotype 4 showed increased infection rates of human lung cells in the presence of a human influenza H3N2 virus but not H1N1 and this effect was significantly higher with the combination ST15063/CC801/GPSC162 lineage from the serotype 4 outbreak and influenza H3N2 virus (Figure 5*C*).

**Figure 5.**
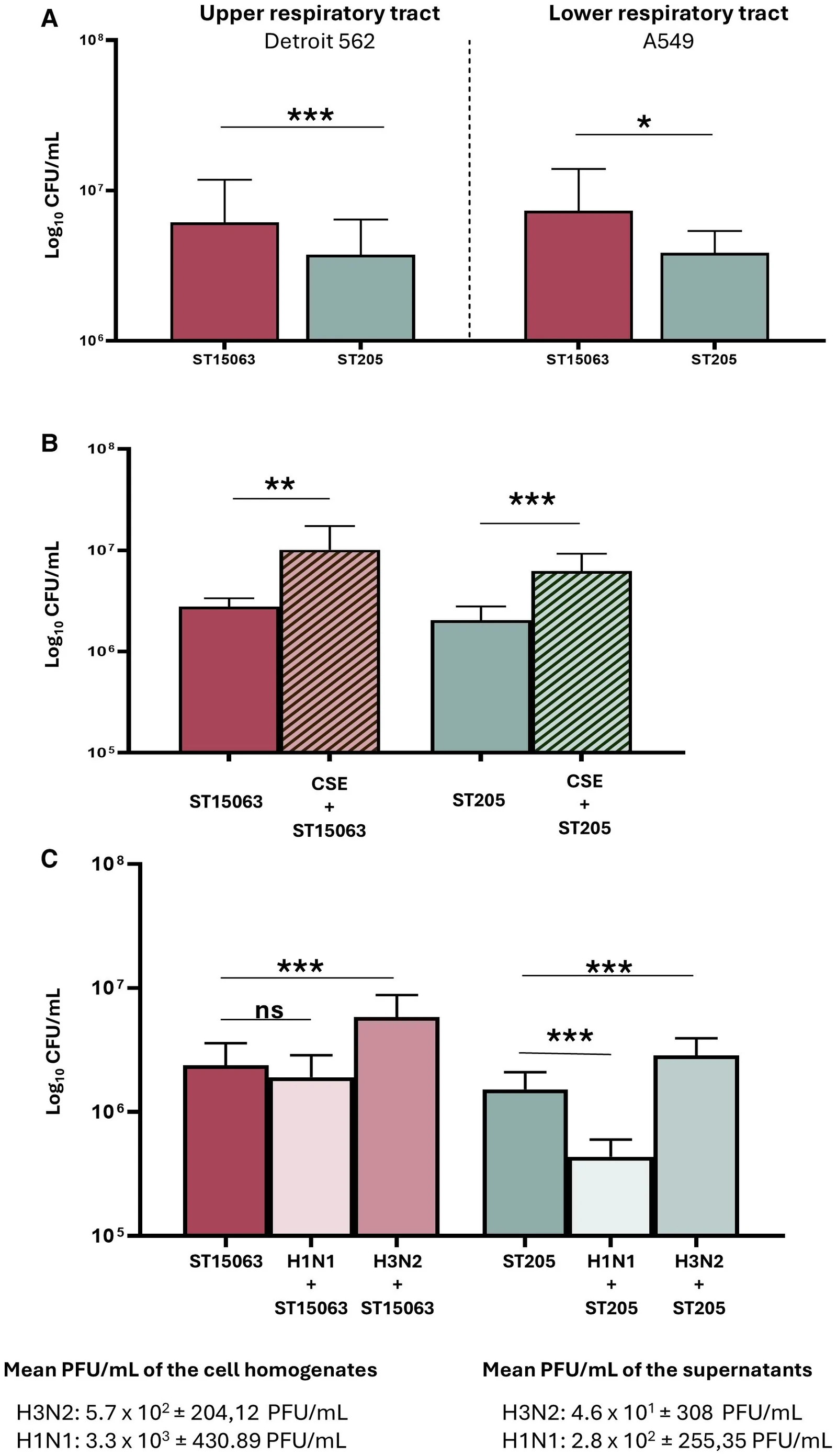
Comparative adhesion of serotype 4 isolates to human lung and nasopharyngeal epithelial cells: contribution of cigarette smoke and influenza infection in CC801 (ST15063, Andalusia outbreak) vs CC205 (ST205). *A*, Adhesion to human nasopharyngeal (Detroit 562) and lung (A549) epithelial cells. *B*, Effect of cigarette smoke exposure on adhesion to lung epithelial cells (A549). *C*, Differences in the effect on secondary pneumococcal adhesion by serotype 4 following primary infection with human influenza A virus (H1N1 and H3N2 variants). Abbreviations: CFU, colony-forming units; CSE, cigarette smoke exposure; ns, not significant; PFU, plaque-forming units; ST, sequence type. Standard deviation bars are shown, and asterisks mark statistically significant results (two-tailed Student’s *t* test: *P < .05; **P < .01; ***P < .001).

## DISCUSSION

Our study documents the unexpected expansion of serotype 4 causing IPD in young adults in Spain after the COVID-19 pandemic and later dissemination to adults ≥65 years, with most cases concentrated initially in the CCAA/region of Andalusia, and particularly in Seville. After the introduction of PCV13 in Spanish children, incidence by serotype 4 has remained low in all populations [21, 22]. Given the low vaccination coverage with PPSV23 or PCV13 among Spanish adults [2, 5], most of the reduction in IPD caused by PCV13 serotypes has been attributed to herd immunity [2, 3]. This specific resurgence of serotype 4 in adults is puzzling because pediatric PCV13 coverage has remained consistently high in Spain (>95%) [5], even during the pandemic, and serotype 4 cases have not upturned in children. Furthermore, studies in the UK have shown that antibody responses to serotype 4 after PCV13 are robust, making it unlikely that the resurgence is due to reduced vaccine-induced herd immunity [8, 23].

The unexpected rise of serotype 4 in young adults has also been observed in other countries. The UK reported an increase in serotype 4 from 24 cases (2019–2020) to 132 cases (2022–2023) and mainly targeted young adults [8]. Moreover, Canada reported an upsurge of IPD cases after COVID-19, observing that serotype 4 was the most prevalent serotype in adults in 2022 (28.4%) [9]. Interestingly, the same region of Canada reported an increase of serotype 4 between 2010 and 2018, particularly among homeless persons, with phylogenetic evidence of both clonal clusters that are in line with outbreak dynamics and more genetically diverse strains disseminating in the community [24]. In addition, the United States (US) has also reported increased proportions of IPD cases by serotype 4 in adults experiencing homelessness [11, 12]. In Northern Europe, similar outbreaks have been described, but in shipyard workers [14], and Belgium has recently confirmed increased rates by serotype 4 [13].

Our findings resemble outbreak dynamics previously described in the US and Canada [11, 12, 24], as we describe an upsurge of cases in a period of 3 years associated with a specific subregion in Spain. Outbreaks of IPD are rare events and are often associated with vulnerable populations and groups living in crowded conditions [25, 26]. Temporal relations are more difficult to establish as pneumococcal outbreaks may occur over a period of several years [24]. Our genomic analysis further supports this interpretation, as the expansion of serotype 4 cases in Seville was almost exclusively driven by ST15063, a lineage within GPSC162 closely related to ST801. Nearly all isolates from Seville formed a tight phylogenetic cluster (3–15 nucleotide differences), strongly suggesting clonal expansion rather than random reintroduction of diverse serotype 4 strains. Moreover, these strains presented a very closed pangenome, indicating high clonality. In contrast, isolates from other Spanish regions were genetically more diverse, encompassing multiple STs within GPSC162 and other lineages such as GPSC27 or GPSC182. This pattern suggests localized amplification of ST15063 in Seville, whereas other regions have a more heterogeneous molecular epidemiology. Moreover, we found the same lineages in serotype 4 IPD affecting adults ≥65 years old, which strengthens the idea that the dissemination of cases to the older populations is caused by the same isolates affecting younger adults.

Association of lineage GPSC162 with outbreaks in other countries has been previously shown, although it was linked to ST801 [14]. In the US and Canada, serotype 4 outbreaks were produced by different STs, including ST244 and ST695 in both countries [12, 24], ST10172 specifically in the US and ST205 in Canada [11, 12, 24]. In Spain, ST244 and ST205 were dominant in the pre-PCV13 period, although cases were scarce and could not be associated with specific populations and geographical location. In the last years, ST247 predominated within GPSC27 after COVID-19, and most isolates did not follow a geographical pattern. In England, despite not correlating serotype 4 cases with clinical data or with certain populations, GPSC162 has also displaced GPSC27 as the main serotype 4 lineage, and IPD cases were driven by ST801 and ST15063 [8, 27]. The reason for the predominance of GPSC162 lineage in serotype 4 in recent years is still unknown. However, we found differences in key virulence factors in GPSC12, such as the absence of PI-1 and a mutation in the main autolysin LytA. PI-1 relevance in pneumococcal pathogenesis is not fully understood, as pili have been associated with colonization, inflammation, and even invasion [28], but the most prevalent serotypes such as 3 and 8 lack both pilus 1 and 2 [6, 7]. The absence of PI-1 in the expanding lineage may suggest alternative mechanisms of host interaction or niche adaptation. Moreover, we found that 99.3% of GPSC162 isolates harbored a mutation in LytA (166His→Tyr). This mutation increases LytA enzymatic activity, and contributes to pathogenesis by increasing lung adhesion, immune evasion, inflammatory response, and virulence in mouse models, which may explain the upsurge of this lineage within serotype 4 [7]. A limitation of the study is that we do not have an explanation for the increased pathogenicity of ST15063 and its predominance in the outburst of cases compared with other STs from the same GPSC. Additionally, we observed that the influenza H3N2 virus increased adherence to the lower respiratory tract, which could explain shared disease patterns between the influenza virus and prevalent pneumococcal serotypes [29]. However, we did not have access to influenza incidence data to demonstrate a shared pattern between serotype 4 and H3N2. The clinical profile of patients suffering IPD in Seville showed that serotype 4 cases were disproportionately associated with substance use as risk factors (including tobacco, alcohol, parenteral drugs, inhaled drugs, and polydrug consumption). In contrast, comorbidities classically linked to IPD [30, 31], such as immunosuppression by any cause or chronic corticosteroid use, were less frequent among serotype 4 patients. This pattern closely mirrors outbreaks in Canada and the US, where serotype 4 disproportionately affected socially vulnerable young adults, often without underlying chronic disease but with high rates of substance use [11, 12, 24]. Specifically, in Canada, serotype 4 cases were also associated with illicit drug use, alcohol abuse, and smoking [24]. In the case of social vulnerability (homelessness and imprisonment), we observed higher proportions in the serotype 4 IPD group (9.4%) than in the IPD group by other serotypes (1.5%), although ratios were lower than those reported in Canada and the US, where approximately 47% and 36% of patients with serotype 4 IPD, respectively, experienced homelessness [11, 12, 24]. In Spain, an estimated 23 000–30 000 people experience homelessness and around 55 000 are imprisoned, together representing only about 1.7% of the population (Instituto Nacional de Estadística, Spain). Taken together, these findings suggest that the expansion of serotype 4 in Seville represents a sociobehaviorally driven outbreak mainly affecting young adults from Seville, rather than waning pediatric herd immunity, facilitated by clonal spread of ST15063. This is a concerning situation because in the year 2024, the rise of serotype 4 started to expand to other Spanish regions, acquiring a national dissemination pattern, and is worrisome because in the last years, we also found an increase of cases affecting adults ≥65 years old.

From a public health perspective, these findings stress the importance of targeted vaccination strategies, considering both direct immunization and adult-to-adult transmission. Despite the availability of new broader vaccines, the 15- and 20-valent PCVs, including serotype 4 in their formulations, adult vaccine uptake in Spain remains low [5]. Experiences from Canada indicate that homelessness and drug use should be considered explicit indications for pneumococcal vaccination, although practical challenges in reaching high vaccine coverage rates in these populations persist [24]. Given the strong association of serotype 4 with behavioral risk factors in Seville, tailored interventions, including vaccination campaigns targeting young adults with substance use, and enhanced outbreak surveillance, are warranted.

In summary, the postpandemic expansion of serotype 4 IPD in Seville represents a clonal outbreak driven by ST15063 within GPSC162, affecting predominantly young adults with high rates of substance use. These findings parallel international experiences of serotype 4 outbreaks in substance users, homeless and occupational groups, stressing the need for better implementation of adult vaccination, and the need for ongoing genomic surveillance to detect and contain future pneumococcal outbreaks.

## Supplementary Material

jiag326_Supplementary_Data

## Supplementary Data

Supplementary materials are available at *The Journal of Infectious Diseases* online (http://jid.oxfordjournals.org/). Supplementary materials consist of data provided by the author that are published to benefit the reader. The posted materials are not copyedited. The contents of all supplementary data are the sole responsibility of the authors. Questions or messages regarding errors should be addressed to the author.
