# Supplementary material for: Lineage Dynamics and Risk Factors Underlying Serotype 4 Invasive Pneumococcal Disease in Spain
Source: J Infect Dis. 2026 Jun 30;234(4):e922–34. doi: 10.1093/infdis/jiag326 (PMC13668353; doi:10.1093/infdis/jiag326)
Supplement: Supplementary file 1 [file jiag326_supplementary_data.zip › alttextforSUPPfigures.docx]

**ALTERNATIVE TEXT FOR SUPPLEMENTARY FIGURES**

SUPPLEMENTARY FIGURE S1

A map of Spain divided by provinces and subregions to highlight specific study areas. The autonomous community and region of Andalusia (AND) in southern Spain is shaded in light pink, encompassing provinces such as Huelva, Cádiz, Córdoba, Málaga, Jaén, Granada, and Almería. Within this region, the province and subregion of Seville (SEV) is explicitly highlighted in bright pink, with an arrow and the text label "SEV" pointing directly to it. The rest of the provinces of Spain remain uncolored in cream, serving as a baseline reference.

SUPPLEMENTARY FIGURE S2

A circular phylogenetic tree of Serotype 4 isolates in Spain, displaying genomes grouped into clades based on single nucleotide polymorphisms (SNPs) against a reference genome. The tree structure radiates from the center, surrounded by multiple concentric metadata rings that annotate each isolate. The innermost ring represents the GPSC lineages, showing a high diversity of clones in the smaller clades (such as GPSC 27, 70, and 182) clustered at the top, while a massive, expanding single clade is uniformly colored in dark red, representing the dominant GPSC 162 lineage. The outer rings denote Subregion, Region, and Epidemiological Period, visually demonstrating that the massive GPSC 162 clade correlates directly with orange highlights for the Seville (SEV) subregion and dark blue segments representing the recent 2022–2024 reopening period.

SUPPLEMENTARY FIGURE S3

A sequence alignment and structural schematic of the LytA autolysin protein (TIGR4 reference, 318 amino acids long) comparing genetic variations across GPSC162, ST247/GPSC27, and other GPSCs. At the top, a nucleotide and amino acid alignment highlights two specific missense mutations: His166Tyr (histidine to tyrosine at position 166) and Gly171Asp (glycine to aspartic acid at position 171). The central diagram displays horizontal bars mapping these mutation sites along the 318-amino-acid length of the protein, showing that GPSC162 carries only the His166Tyr mutation, while ST247/GPSC27 carries both mutations. At the bottom, two color-coded multi-sequence alignments illustrate the localized impact of these substitutions on protein Hydrophobicity and Polarity from amino acids 160 to 172, visually showing the biophysical alterations introduced by the tyrosine (Tyr) and aspartic acid (Asp) residues compared to the TIGR4 reference.

SUPPLEMENTARY FIGURE S4

Two panels (A and B) analyzing the Serotype 4 pangenome composition and gene accumulation curves across major lineages. Panel A focuses on overall Serotype 4 isolates divided by GPSC (GPSC162, GPSC27, GPSCNA, and GPSC70). It features a stacked bar chart showing the proportion of core genes (dark red), shell genes (pink), and cloud genes (blue), with a thick vertical blue line highlighting the accessory genome; alongside an accumulation curve plotting the number of gene differences against the number of genomes, where the overall isolates curve continues to rise while individual GPSC lines scale lower. Panel B replicates this analysis specifically within the GPSC162 lineage, comparing ST15063 against other STs. It shows that ST15063 has a highly conserved pangenome dominated by core genes (nearly 90%) and a significantly flatter gene accumulation curve compared to other STs, indicating a closed and highly uniform clonal structure.

SUPPLEMENTARY FIGURE S5

Two bar charts comparing the variability in respiratory adhesion to human epithelial cells across four distinct pneumococcal genotypes (ST15063, ST801, ST13022, and ST205). The y-axis measures bacterial adhesion in Log10 CFU/mL. Panel A displays adhesion to upper respiratory tract cells (Detroit 562), showing that the Spain outbreak clone ST15063 exhibits the highest mean adhesion capacity, being significantly higher than ST801, ST13022, and ST205. Panel B illustrates adhesion to lower respiratory tract cells (A549), where ST15063 maintains elevated adhesion levels that are significantly greater than both ST13022 and ST205, while showing no statistically significant difference compared to the Northern European outbreak clone ST801.
