# Supplementary material for: Lineage Dynamics and Risk Factors Underlying Serotype 4 Invasive Pneumococcal Disease in Spain
Source: J Infect Dis. 2026 Jun 30;234(4):e922–34. doi: 10.1093/infdis/jiag326 (PMC13668353; doi:10.1093/infdis/jiag326)
Supplement: Supplementary file 1 [file jiag326_supplementary_data.zip › Supplementary Figures S1-S5.pdf]

SUPPLEMENTARY FIGURE S1

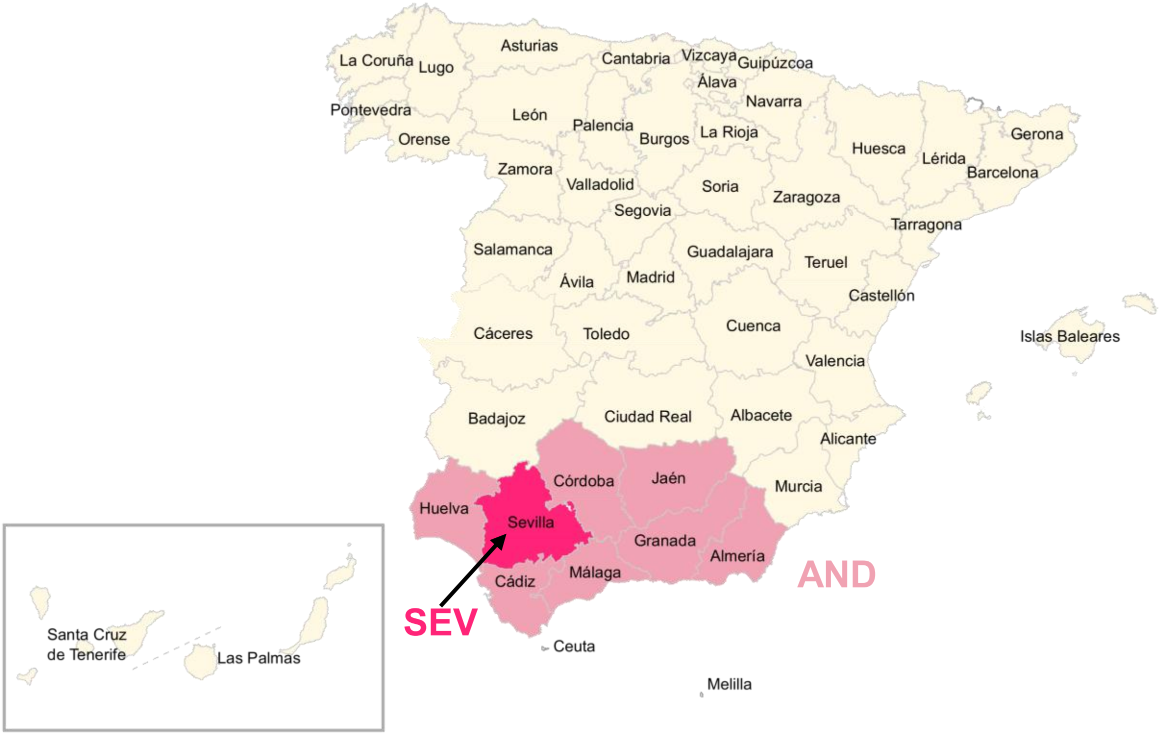

Supplementary Figure S1. Map of Spain divided in provinces/subregions. The CCAA/region of Andalusia (AND) is highlighted in pink, and the province/subregion of Seville (SEV) within AND is highlighted in bright pink.

SUPPLEMENTARY FIGURE S2

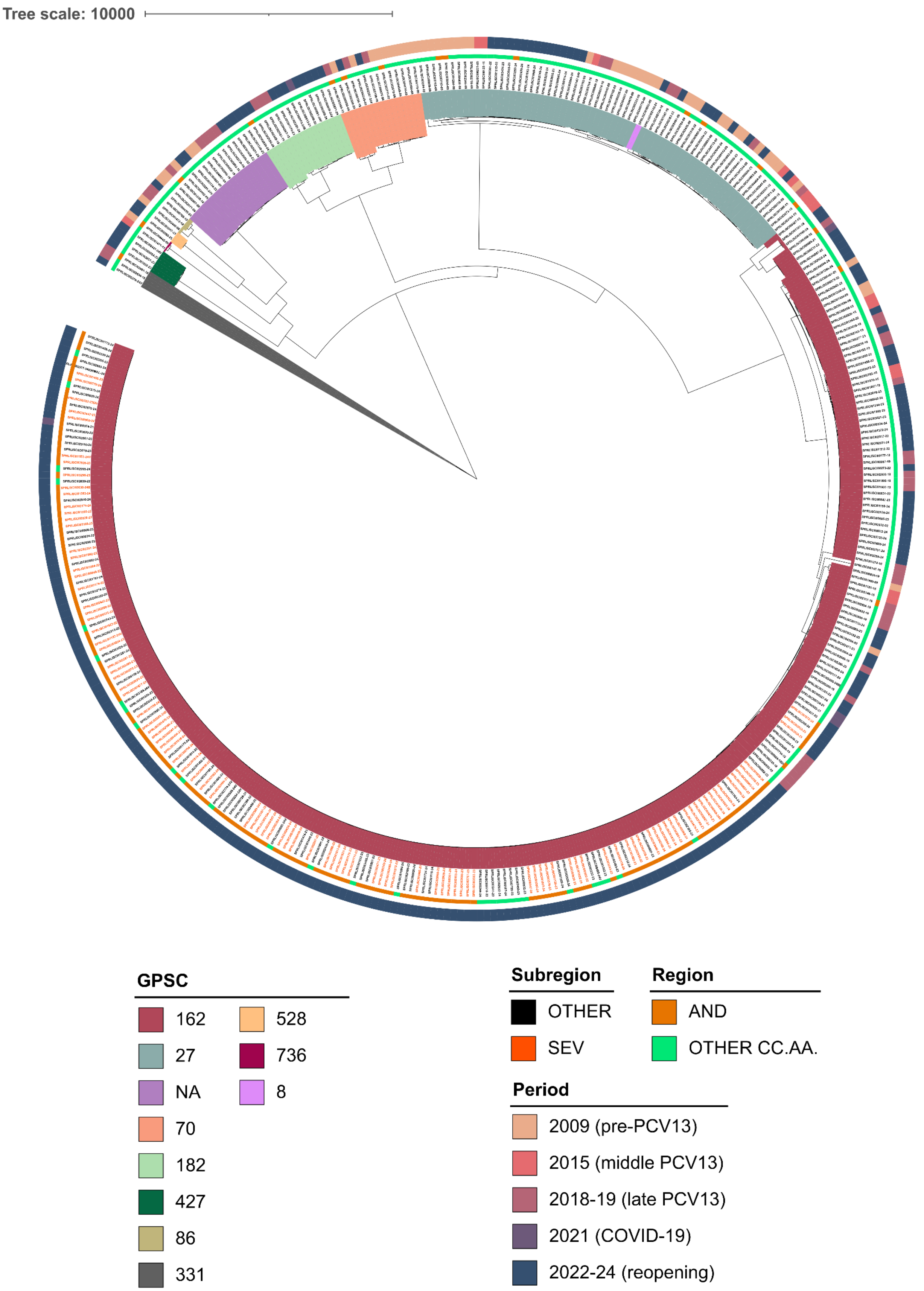

Supplementary Figure S2 Phylogenetic tree of serotype 4 in Spain. The phylogenetic tree was obtained using the snippy, snippy-core, and Gubbins steps described in methods, and groups genomes into different clades based on the SNPs against the reference (GCA_09128732). Geographic region was depicted (AND vs Other Spanish regions), and Seville isolates are highlighted in orange. Annotated data is described in the legend.

SUPPLEMENTARY FIGURE S3

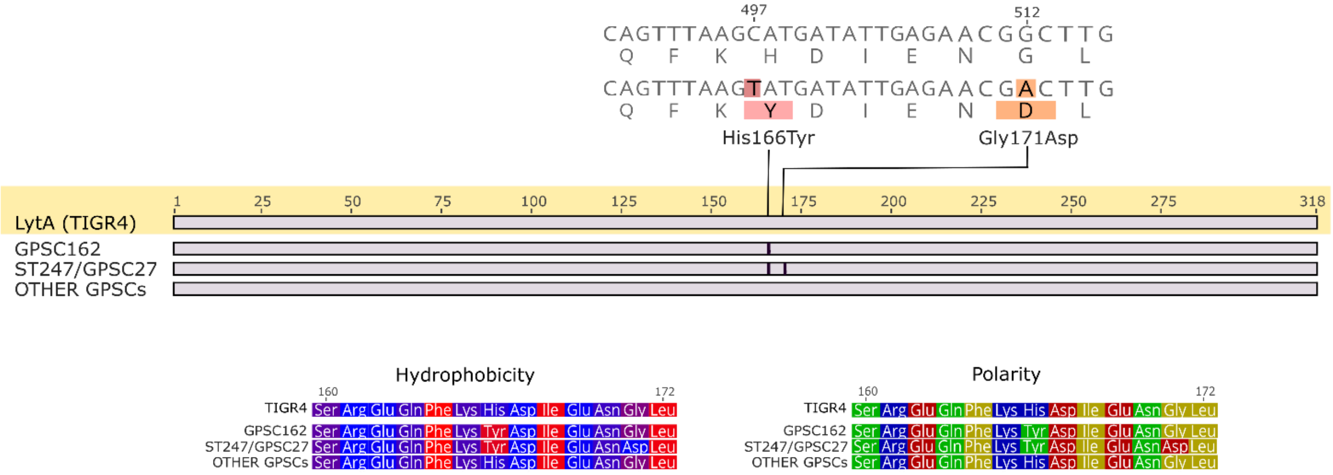

Supplementary Figure S3. Differences in LytA between serotype 4 main lineages. Protein alignment of LytA proteins from different serotype 4 isolates against the NAM-amidase of TIGR4 as reference (WP_000405234.1). Hydrophobicity (from red through blue, being red most hydrophobic) and polarity (non-polar in yellow, polar-uncharged in green, polar-acidic in red, and polar-basic in blue) changes in the 160–172 region of the protein are shown.

SUPPLEMENTARY FIGURE S4

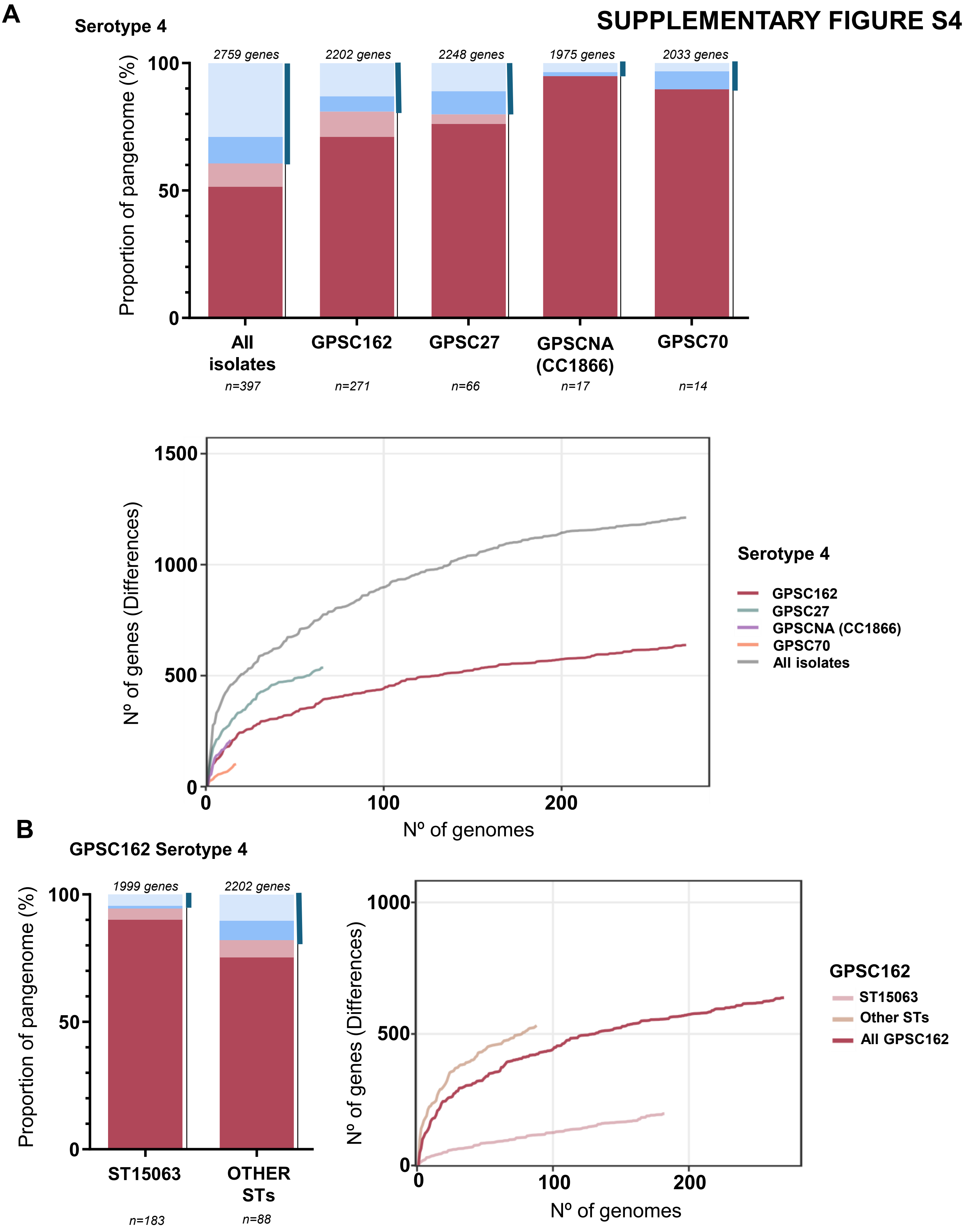

Supplementary Figure S4. Analysis of the serotype 4 pangenome and major lineages. Percentage of core and accessory genome and relationship analysis between the total and conserved genes of overall serotype 4 isolates and the major lineages. The X-axis shows the number of genomes and the Y-axis the difference between the total and conserved genes in the pangenome analysis for each added genome (A). Percentage of core and accessory genome and relationship analysis between the total and conserved genes of overall GPSC162 isolates and ST15063 (B). Thick blue line highlights the proportion of the accessory genome (shell and cloud genes). GPSC not assigned (GPSCNA).

SUPPLEMENTARY FIGURE S5

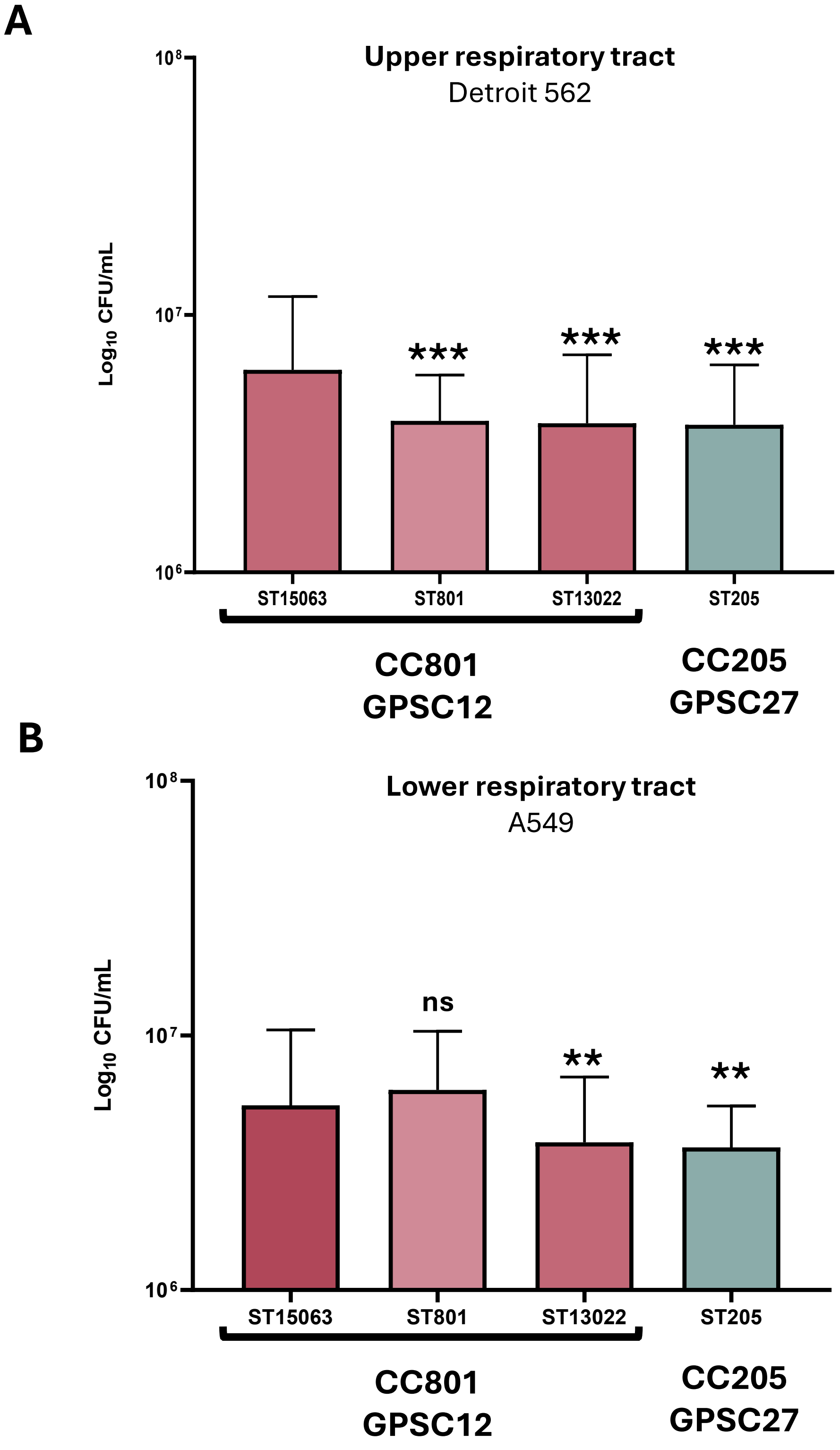

Supplementary Figure S5. Variability in respiratory adhesion to the upper (A) and lower (B) respiratory tract between genotypes within serotype 4. Adhesion to human nasopharyngeal (Detroit 562) and lung (A549) epithelial cells. We included CC801/GPSC162 isolates (ST15063 responsible for the outbreak in Spain, ST801 responsible for the outbreak in Northern Europe, and ST13022), as well as CC205/GPSC27 (ST205 responsible for the outbreak in Canada). Error bars represent the standard deviation, and asterisks indicate statistically significant results (two-tailed Student's t-test: $P < 0.05$, $P < 0.01$, *** $P < 0.001$); ns, non-significant
