# Supplementary material for: Lineage Dynamics and Risk Factors Underlying Serotype 4 Invasive Pneumococcal Disease in Spain
Source: J Infect Dis. 2026 Jun 30;234(4):e922–34. doi: 10.1093/infdis/jiag326 (PMC13668353; doi:10.1093/infdis/jiag326)
Supplement: Supplementary file 1 [file jiag326_supplementary_data.zip › SupplementaryMethods.docx]

**Lineage dynamics and risk factors underlying serotype 4 invasive pneumococcal disease in Spain**

**Supplementary Methods**

**Study design**

We characterized IPD isolates affecting all age groups submitted by hospital microbiology laboratories to the Spanish Pneumococcal Reference Laboratory (SPRL). The SPRL notifies every year to the European Center for Disease Control (ECDC) all IPD cases in Spain and to the Invasive Respiratory Infection Surveillance Network (IRIS) [1, 2]. The epidemiological year considered in the manuscript is from January to December.

Due to the specific location of serotype 4 cases in Spain, we also represent cases by geographical regions (Spanish Autonomous Communities or CCAA). Spain is divided in 17 autonomous communities (CCAA) or regions and 2 autonomous cities. These CCAA/regions are divided into different subregions known as provinces. Andalusia (AND) is an CCAA/region in southern Spain, and Seville (SEV) is a province/subregion of Andalusia (Supplementary Figure S1).

PCV13 was initially approved for pediatric vaccination in Spain in 2010 as private use although reaching 67–82% pediatric coverage [3]. In 2016, it was included into the national pediatric schedule (2+1 regimen) and used in some regions for immunocompetent adults ≥65 years and also for high-risk adults, following a sequential PCV13+PPV23 schedule. Pediatric coverage remained high (95–97%) since 2016, even during the pandemic, while adult coverage was low (PCV13: 23–29%, PPV23: 18–26%). Throughout 2024, some regions changed to PCV15 or PCV20 for children and PCV20 for immunocompetent adults.

**Whole genome sequencing and bioinformatic analysis**

We performed an in-deep WGS analysis of 397 serotype 4 clinical isolates from different vaccine periods: pre-PCV13 (n=51), middle PCV13 (n=15), late PCV13 (n=47), COVID-19 (n=5), and reopening including the last epidemiological year (n=279) (Supplementary Table 1). We sampled a strong representation of more than 60% of serotype 4 isolates for the period 2009-2024. Isolates were selected using a randomization approach to ensure representative temporal sampling and to minimize selection bias. Of all isolates tested, 17 belonged to the pediatric population, 58 to adults ≥65 years, and the majority to young adults (18-64 years old), as they had the highest burden of disease by serotype 4 (n=322) (Supplementary Table S1). Isolates were selected using a randomization approach in order to ensure representative temporal sampling and to minimize selection bias.

Chromosomal DNA was extracted using the Wizard Genomic DNA Purification Kit (Promega). Sequencing libraries were prepared with the Illumina DNA Prep (96) kit and IDT for Illumina Nextera DNA Unique Dual Indexes, followed by paired-end sequencing (2 × 150 bp) on a NovaSeq 6000 platform at the Genomics Unit, ISCIII. The resulting reads have been deposited in the European Nucleotide Archive (Supplementary Table S1). WGS analysis was conducted using the Bactopia Nextflow pipeline [4]. The workflow included quality assessment with FASTQC (<https://www.bioinformatics.babraham.ac.uk/projects/fastqc/>), removal of low-quality reads, decontamination from non-target bacterial sequences, and de novo genome assembly using Shovill (<https://github.com/tseemann/shovill>). Bactopia integrates multilocus sequence typing (MLST; <https://github.com/tseemann/mlst>) and genome annotation with Prokka (<https://github.com/tseemann/prokka>) without additional processing. Pneumococcus-specific analyses were performed with PBP-typer (<https://github.com/rpetit3/pbptyper>). Preliminary screening for antimicrobial resistance (AMR) genes and virulence factors was carried out using Abricate (<https://github.com/tseemann/abricate>). For phylogenetic analysis, single-nucleotide polymorphism (SNP) calling was performed with Snippy (<https://github.com/tseemann/snippy>), phylogenetic trees were inferred using RAxML-NG (<https://github.com/amkozlov/raxml-ng>), and recombination events were identified with Gubbins (<https://github.com/nickjcroucher/gubbins>). All isolates were classified in sequence-type (ST) based upon the 7-locus multilocus sequence typing (MLST) scheme. CCs cluster pneumococci based upon the ST [5]. GPSCs define pneumococcal clusters based upon the analysis of core and accessory genes [6]. For serotype 4 phylogenetic analysis, reads were mapped to the ST801/CC801/GPSC162 reference genome described by Gladstone et al. (2022) (GCA_09128732) [7]. The reference was prepared in Geneious Prime (Biomatters) by removing contigs <500 bp, reordering the remaining contigs with Mauve alignment against the complete ATCC700669 genome, and concatenating them into a final sequence of 2,051,094 bp.

Geneious R9 (Biomatters) was also used to investigate mutations and gene acquisitions associated with AMR and to examine major virulence factors in serotype 4 isolates. Virulence determinants were identified using reference annotations from the TIGR4, D39, R6, OXC141, and ATCC700669 genomes. Prophages were classified into PPH families [8], and integrative and conjugative elements (ICEs) were detected using ICEberg 3.0 (<https://tool2-mml.sjtu.edu.cn/ICEberg3/>). Global phylogenetic contextualization and GPSC lineage assignments were obtained from PathogenWatch (<https://pathogen.watch/>).

**Clinical data from patients hospitalized with IPD**

Due to the specific expansion of serotype 4 causing IPD among young adults in the subregion of Seville, (Andalusia, Spain) since 2022, we evaluated clinical and demographic data of young adults (18–65 years) diagnosed with IPD by any serotype between January 2022 and December 2024 from the five main hospitals in Seville: Hospital Universitario Virgen del Rocío, Hospital Universitario Virgen de Macarena, Complejo Hospitalario Nuestra Señora de Valme, Hospital San Juan de Dios del Aljarafe, and Hospital de la Merced.

Demographic variables included age, sex, postal code type (categorized as: home address with valid postal code, street homelessness, or penitentiary). Substance consumption included smoking status, alcohol, parenteral drug use, inhaled drug use and polydrug use. Comorbidities comprised chronic obstructive pulmonary disease (COPD), asthma, severe COVID-19, other chronic respiratory diseases, HIV infection, immunosuppression (of any cause), chronic corticosteroid use, arterial hypertension, diabetes mellitus, and dyslipidemia. Vaccination status was recorded for both COVID-19 and pneumococcal vaccines (categorized as no vaccination, PCV13, or PCV13+PPV23). Mortality during hospital admission (exitus) was also recorded.

**Infection of human nasopharyngeal and lung epithelial cells**

Experiments involving infection assays with Detroit 562 (nasopharyngeal cells) and A549 (lung cells) were performed using different pneumococcal strains of serotype 4 from young adults (18-65 years old) and influenza isolates (Supplementary Table S2).

Cigarette smoke extract (CSE) was obtained from research cigarettes from University of Kentucky (nicotine 0.73 mg, tar 9.4 mg, CO 12 mg) using a modified syringe-based dispositive to bubble cigarette smoke into 5 mL sterile RPMI-1640 medium per cigarette. The stock solution of CSE was obtained from the combustion of eight cigarettes and it was defined as 100% CSE, aliquoted, and stored at −80 °C as previously described [10].

Infection assays of human nasopharyngeal and lung epithelial cells were performed with Detroit 562 /A549 cells (2×10⁵) cultured in RPMI in the presence or absence of 5% CSE and infected for 1 h with pneumococcal strains (MOI 25). After washing, cells were homogenized, and viable bacteria were quantified (CFU/mL).

For coinfection assays of *S. pneumoniae* and influenza virus, these experiments were performed using the A549 cell line cultured in DMEM supplemented with 0.2% BSA, 1 mM MgCl₂, and 0.9 mM CaCl₂. Briefly, 2×10⁵ cells were infected at a multiplicity of infection (MOI) of 0.5 with influenza A/New Caledonia/20/1999 (H1N1 subtype) or influenza A/Darwin/9/2021 (H3N2 subtype). After 1 h of viral adsorption, the infection medium was removed and cells in 500 µL of DMEM supplemented were incubated for 24 h at 37 °C with 5% CO₂. The following day, cells were infected with *Streptococcus pneumoniae* (strains 582 [ST15063/CC801/GPSC162] or 724 [ST205/CC205/GPSC27]) at an MOI of 10. After 1 h of bacterial adhesion, the supernatant was removed and cells were homogenized. This homogenate was used to determine pneumococcal viable counts (CFU/mL) and to confirm influenza infection (PFU/mL). Viral titers in supernatants were determined by indirect immunofluorescence staining of infected MDCK cells (female origin Madin-Darby canine kidney cells, ATCC CRL-3216) as described previously [9].

**Statistical analysis**

The corrected incidences were calculated as the number of IPD episodes per 100,000 population and year using population data from the Spanish National Statistical Institute and the Institute of Statistics of Andalusia as the denominator and considering the population capture of 80% (4). We assumed the same epidemiological characteristics for the population suffering from IPD covered by hospitals and the general population. Comparison of different periods was analyzed by calculating the IRRs using Poisson regression models. Statistical analyses were performed using STATA v.14.

For the clinical data statistical analysis, the primary comparison was between patients infected by serotype 4 versus patients infected by all other serotypes. Categorical variables between groups were compared using Pearson’s Chi-squared test when all expected cell counts were high, and Fisher’s exact test was applied if Chi-square test assumptions were violated. All analysis were performed in R version 4.3.2.

All experiments were performed in at least three independent replicates. Statistical analyses were conducted using two-tailed Student’s t-test or one-way ANOVA with Dunnett’s post hoc test, with significance defined as P < 0.05 (*), P < 0.01 (**), and P < 0.001 (***).

**REFERENCES**

1. Shaw D, Abad R, Amin-Chowdhury Z, et al. Trends in invasive bacterial diseases during the first 2 years of the COVID-19 pandemic: analyses of prospective surveillance data from 30 countries and territories in the IRIS Consortium. Lancet Dig Health **2023**; 5:e582-e93.

2. Shaw D, Torreblanca RA, Amin-Chowdhury Z, et al. The importance of microbiology reference laboratories and adequate funding for infectious disease surveillance. Lancet Dig Health **2025**; 7:e275-e81.

3. Pérez-García C, Sempere J, de Miguel S, et al. Surveillance of Invasive Pneumococcal Disease in Spain Exploring the Impact of the COVID-19 Pandemic (2019-2023). J Infect **2024**:106204.

4. Petit RA, 3rd, Read TD. Bactopia: a Flexible Pipeline for Complete Analysis of Bacterial Genomes. mSystems **2020**; 5.

5. Feil EJ, Li BC, Aanensen DM, Hanage WP, Spratt BG. eBURST: inferring patterns of evolutionary descent among clusters of related bacterial genotypes from multilocus sequence typing data. J Bacteriol **2004**; 186:1518-30.

6. Gladstone RA, Lo SW, Lees JA, et al. International genomic definition of pneumococcal lineages, to contextualise disease, antibiotic resistance and vaccine impact. EBioMedicine **2019**; 43:338-46.

7. Gladstone RA, Siira L, Brynildsrud OB, et al. International links between *Streptococcus pneumoniae* vaccine serotype 4 sequence type (ST) 801 in Northern European shipyard outbreaks of invasive pneumococcal disease. Vaccine **2022**; 40:1054-60.

8. Martin-Galiano AJ, Garcia E. *Streptococcus pneumoniae*: a Plethora of Temperate Bacteriophages With a Role in Host Genome Rearrangement. Front Cell Infect Microbiol **2021**; 11:775402.

9. Diego J-B, Arranz-Herrero J, Laghlali G, et al. Disease modulation by TIV vaccination during secondary pneumococcal infections in influenza-infected mice. J Virol **2025**:e0177425.

10. Martí-Lliteras P, Regueiro V, Morey P, et al. Nontypeable Haemophilus influenzae clearance by alveolar macrophages is impaired by exposure to cigarette smoke. Infect Immun. 2009 Oct;77(10):4232-42.
